# Ti_3_Si_0.75_Al_0.25_C_2_ Nanosheets as Promising Anode Material for Li-Ion Batteries

**DOI:** 10.3390/nano11123449

**Published:** 2021-12-20

**Authors:** Jianguang Xu, Qiang Wang, Boman Li, Wei Yao, Meng He

**Affiliations:** School of Materials Science and Engineering, Yancheng Institute of Technology, Yancheng 224051, China; wangqiang970808@sina.com (Q.W.); liboman0919@sina.com (B.L.); weiyao@ycit.edu.cn (W.Y.)

**Keywords:** MAX phases, ultrathin nanosheets, lithium-ion battery, electrochemical performance, anode materials

## Abstract

Herein we report that novel two-dimensional (2D) Ti_3_Si_0.75_Al_0.25_C_2_ (TSAC) nanosheets, obtained by sonically exfoliating their bulk counterpart in alcohol, performs promising electrochemical activities in a reversible lithiation and delithiation procedure. The as-exfoliated 2D TSAC nanosheets show significantly enhanced lithium-ion uptake capability in comparison with their bulk counterpart, with a high capacity of ≈350 mAh g^−1^ at 200 mA g^−1^, high cycling stability and excellent rate performance (150 mAh g^−1^ after 200 cycles at 8000 mA g^−1^). The enhanced electrochemical performance of TSAC nanosheets is mainly a result of their fast Li-ion transport, large surface area and small charge transfer resistance. The discovery in this work highlights the uniqueness of a family of 2D layered MAX materials, such as Ti_3_GeC_2_, Ti_3_SnC_2_ and Ti_2_SC, which will likely be the promising choices as anode materials for lithium-ion batteries (LIBs).

## 1. Introduction

Since they were first reported, lamellar ternary carbides and nitrides have been named “MAX phases” or “M_n+1_AX_n_ phases (*n* = 1, 2 or 3)”, where M represents an early transition metal, A is an element of ⅢA to VIA groups and X is carbon or nitrogen. They have attracted great attention because of their special combination of metallic and ceramic properties [1]. For example, due to their inherent layered structure with alternately arranged MX and A layers, the MAX phases display a superior resistance to oxidation, thermal energy and corrosion, very good electrical conductivity, high strength and elastic modulus and excellent machinability [2,3,4,5,6]. Owing to their lamellar structure and excellent conductivity, MAX phases show great potential in lithium-ions storage for a Li-ion battery (LIB) or capacitor [7,8,9,10,11,12,13,14]. However, the reported capacities of MAX phases are relatively low, particularly in the initial few charge–discharge cycles, which may restrict their real application in LIB. Thus, it is important to optimize the lithium-ion uptake property of MAX phases. It has been well accepted that the nanoscale materials, particularly the ultrathin two-dimensional (2D) nanosheets, have improved properties compared to their corresponding bulk counterparts [15,16,17,18]. For instance, the reversible capacity of free-standing graphene nanosheet (GNS) was found to be 540 mAh g^−1^ [19], and that of N-GNS even reached a high value of 684 mAh g^−1^ [20], both of which are over the theoretical reversible capacity of graphite. In that case, it is anticipated that MAX nanosheets can exhibit an enhanced lithium-ion storage property compared to their bulk materials.

Unfortunately, unlike the inorganic graphene analog (IGA) with weak van der Waals force between its layers, the MAX phases have relatively robust connections between the MX and A layers, and it seems difficult to exfoliate the bulk MAX materials into ultrathin nanosheets by a facile sonic exfoliation process [21]. Particularly, most of the Ti_3_SiC_2_ and Ti_2_SC particles were broken into small species instead of being delaminated to ultrathin nanosheets by increasing the power of sonication [10]. To overcome this limitation, we developed an available substitutional-solid-solution-based exfoliation process for the large-scale fabrication of ultrathin nanosheets of A-layer-activated MAX phases. As a result, ultrathin Ti_3_Si_0.75_Al_0.25_C_2_ (TSAC) nanosheets with a very thin thickness of 4 nm were prepared based on this strategy, which can be used as a promising filler for polymer composites [22,23].

**Figure 1:**
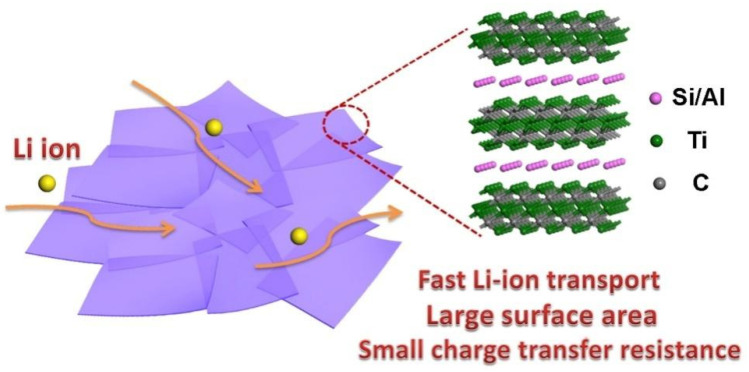
Schematic illustration of the advantages by using TSAC nanosheets-based electrode for Li-ion battery.

Herein, we extend the application of these TSAC nanosheets to the electrode for LIBs because the size and morphology of MAX phases promise improvements on their electrochemical performance for LIBs [9,10,11]. The Ti_3_Si_0.75_Al_0.25_C_2_ nanosheets have very thin thickness, so it can be expected that Li ions can easily be intercalated into the TSAC nanosheets layers. In addition, the electrical conductivity of a MAX phase is normally higher than its MXene counterpart, which usually has excellent conductivity [24], because the A-group layer increases the metallic properties of the material for the improvement of their overall electrochemical properties [1]. Hence, per the diagram in Figure 1, combining the large surface area and excellent conductivity with Ti_3_Si_0.75_Al_0.25_C_2_ nanosheets, high performance can be anticipated for the Ti_3_Si_0.75_Al_0.25_C_2_ nanosheets electrode.

## 2. Experimental Section

### 2.1. Materials

Titanium powder (300 mesh, 99.9 wt.%) was purchased from Guangzhou metallurgy (Guangzhou, China). Silicon powder (200 mesh, 99.0 wt.%), aluminum (Al) powder (200 mesh, 99.0 wt.%), graphite powder (30 μm, 99.85 wt.%) and absolute alcohol (AR) were purchased from Sinopharm (Shanghai, China).

### 2.2. Preparation of Ti_3_Si_0.75_Al_0.25_C_2_ Powder

The Ti_3_Si_0.75_Al_0.25_C_2_ powder (bulk TSAC) was synthesized via a self-propagation high-temperature synthesis (SHS) process. In detail, 14.3 g Ti powder, 4.21 g Si powder, 0.68 g Al powder and 2.4 g graphite powder were blended using a QM-BP Ball Mill at 300 rpm for 2 h, in which the molar ratio of Ti: Si: Al: C was about 3:1.5:0.25:2. The as-received mixture was then put into a self-propagation high-temperature reactor, and then ignited by a tungsten filament under the protection of pure Ar gas. After combustion, a gray product was collected for further processing. The atomic ratio of the Ti, Si, Al and C of the as-received powder is about 3:0.75:0.25:2, which was determined by X-ray fluorescence spectroscopy (XRF) in our previous work [22].

### 2.3. Preparation of Ultrathin Ti_3_Si_0.75_Al_0.25_C_2_ Nanosheets

The Ti_3_Si_0.75_Al_0.25_C_2_ nanosheets (TSAC nanosheets) were obtained by the liquid exfoliation of TSAC powder in absolute alcohol via sonication. In detail, 2 g bulk TSAC powder was dispersed in 0.2 L absolute alcohol, and then sonicated for 24 h. After sonication, the resulting dispersion liquid was then centrifuged at 2000 rpm for 20 min to remove most residual large-size particles. Finally, TSAC nanosheets were dried and collected for the following tests through vacuum filtration of the resulting supernatant.

### 2.4. Preparation of Electrodes

The electrochemical behaviors of TSAC nanosheets and bulk TSAC were studied using CR-2032 coin-cell with lithium metal as the counter electrode and reference electrode. The batteries were based on Li metal (−) | | TSAC (+) with liquid electrolyte (1M solution of LiPF_6_ in ethyl carbonate (EC)-dimethyl carbonate (DMC)-ethyl methyl carbonate (EMC) (1:1:1, *v*/*v*/*v*)). Microporous polypropylene membrane (Celgard2500) was used as a separator. Next, 80 wt.% TSAC nanosheets or bulk TSAC, 10 wt.% acetylene black and 10 wt.% polyvinylidene fluoride (PVDF) were dispersed in N-methylpyrrolidone (NMP) and uniformly mixed into a viscous slurry. Then, the slurry was deposited on a copper foil current collector. The electrodes were then vacuum dried for 12 h at 120 °C, followed by electrochemical evaluation. The loading of active material was about 0.65~0.85 mg cm^−2^. Finally, the cells were assembled in a glove box filled with 99.99 wt.% Ar gas.

### 2.5. Characterization

The TSAC powder was characterized by X-ray powder diffraction (XRD) with a Japan Rigaku Dmax X-ray diffractometer (Tokyo, Japan) equipped with graphite monochromatized high-intensity Cu-Kα radiation (λ = 1.54178 Å). The field emission scanning electron microscopy (FE-SEM) images were performed using a FEI Nova NanoSEM 450 scanning electron microscope (Hillsboro, OR, USA). The transmission electron microscopy (TEM) images were taken on a JEM-2100F field emission electron microscope (Tokyo, Japan) with X-MaxN 80T IE250 Energy Disperse Spectroscopy (UK). The Brunauer–Emmett–Teller (BET) specific surface areas of the samples were determined using a Micromeritics TriStar II 3020 system (Norcross, GA, USA). X-ray photoelectron spectroscopy (XPS, Thermo Fisher ESCALAB 250Xi, Waltham, MA, USA) was utilized to explore the electron-binding energy of TSAC nanosheets. The Raman spectra of the samples were recorded by a Renishaw inVia Reflex system (Gloucestershire, UK) equipped with an argon ion Laser with a wavelength of 633 nm. The cells were charged and discharged galvanostatically in a fixed voltage window from 0.001 to 3 V on a Shenzhen Neware battery cycler (Shenzhen, China) at room temperature. All the gravimetric capacity data related to as-prepared samples were based on the mass of TSAC nanosheets or bulk TSAC. Cyclic voltammetry and electrochemical impedance spectroscopy (EIS, frequency range: 0.1–10^5^ Hz, amplitude: 5 mV) analysis were carried out by a Zahner-Zennium electrochemical workstation (Kronach, Germany).

## 3. Results and Discussion

**Figure 2:**
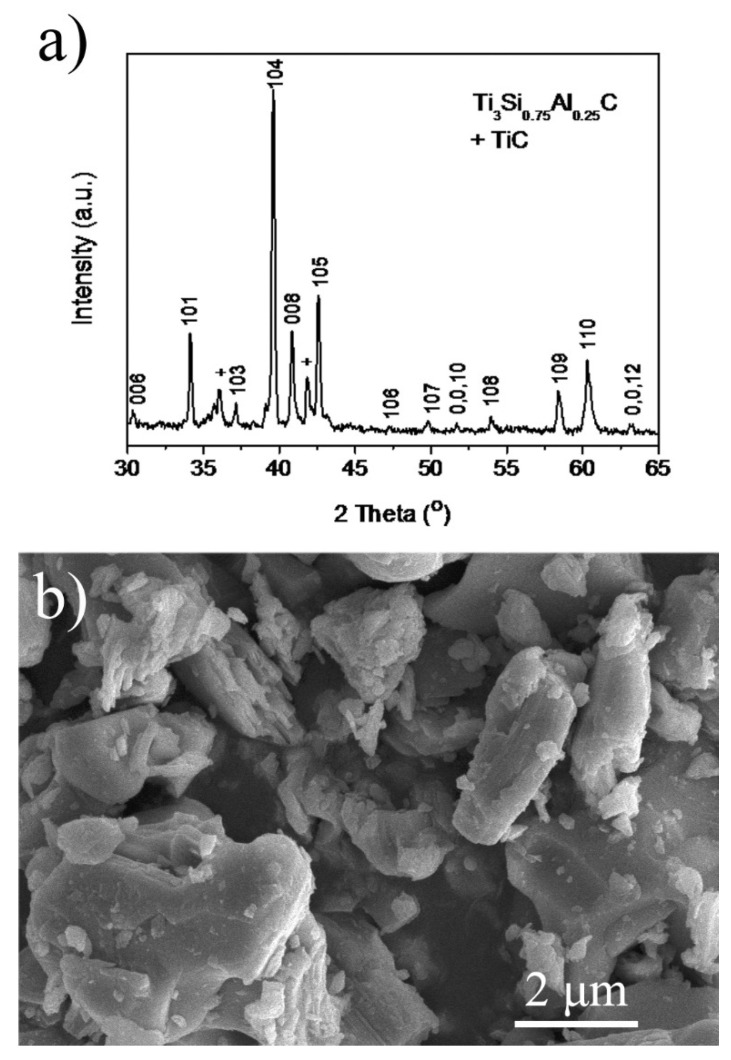
(**a**) XRD pattern and (**b**) SEM micrograph of the product synthesized by SHS.

The feasibility of using bulk TSAC and exfoliated TSAC nanosheets as electrodes for LIBs was investigated. The XRD pattern and SEM image of a bulk TSAC are shown in Figure 2. The primary phase of this product is very close to the pattern of Ti_3_SiC_2_ (JCPDS card No. 89-1356). A small blue shift also appears (Figure 2a), indicating the formation of a solid solution Ti_3_Si_0.75_Al_0.25_C, as discussed in our previous work [22]. In addition, a small amount of TiC is also found in the XRD pattern of bulk TSAC, in most situations which is generated with the formation of Ti_3_SiC_2_ [25]. The micrograph of the product (Figure 2b) shows a distinct lamellar structure, which is in agreement with the crystal structure of Ti_3_SiC_2_. As shown in Figure 1, the crystal structure of Ti_3_SiC_2_ is composed of Ti_3_C_2_ layer and Si layer along *c*-direction. Because the connection between the Ti_3_C_2_ layer and the Si layer is relatively low and can be exfoliated, Ti_3_SiC_2_ and other MAX phase compounds generally display a lamellar appearance.

**Figure 3:**
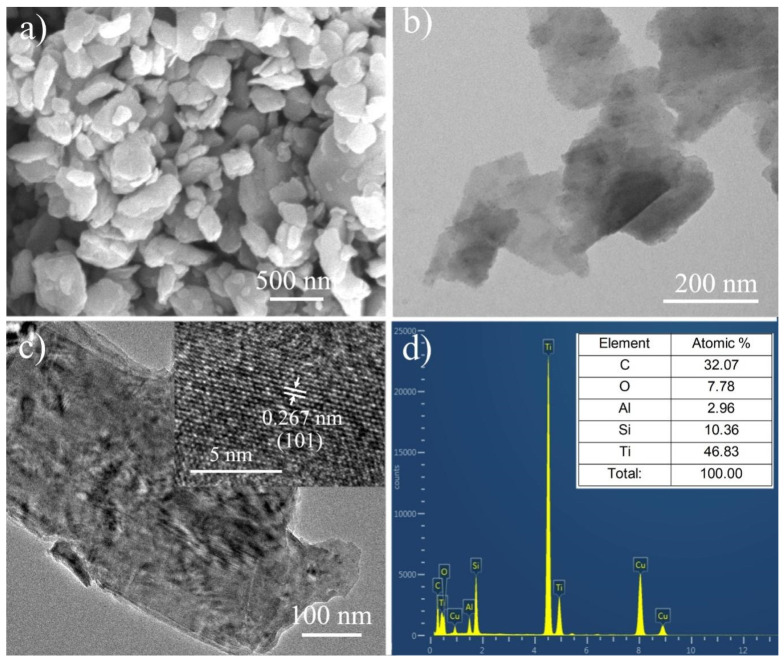
(**a**) SEM and (**b**,**c**) TEM images of the exfoliated TSAC nanosheets. (**d**) Energy-dispersive X-ray spectroscopy (EDX) analysis of TSAC nanosheets, performed on the center of the nanosheet in Figure 3c.

The microstructure of the TSAC nanosheets is shown in Figure 3. The sample exhibits a sheet-like morphology with a size from around 100 to 1000 nm. According to a TEM image of the nanosheets (Figure 3b), most of them exhibit ultrathin sheet-like structures, which render them transparent or semitransparent. Moreover, it could be detected from a magnified nanosheet in Figure 3c that TSAC nanosheets are composed of only a few layers, indicating that bulk TSAC has been delaminated successfully and the crystal structure of Ti_3_Si_0.75_Al_0.25_C_2_ (inserted in Figure 3c) is well maintained during sonication. The BET results further confirmed the exfoliated sheet-like structure. Compared with the bulk TSAC, the specific surface area (SSA) of the TSAC nanosheets increased from 4.25 m^2^ g^−1^ to 11.68 m^2^ g^−1^. In addition, on the basis of EDX analysis in Figure 3d, the atomic ratios of Ti:(Si + Al):C and Si:Al are close to 3:1:2 and 3:1, respectively, suggesting that the composition of Ti_3_Si_0.75_Al_0.25_C_2_ particles has no obvious change after delamination.

**Figure 4:**
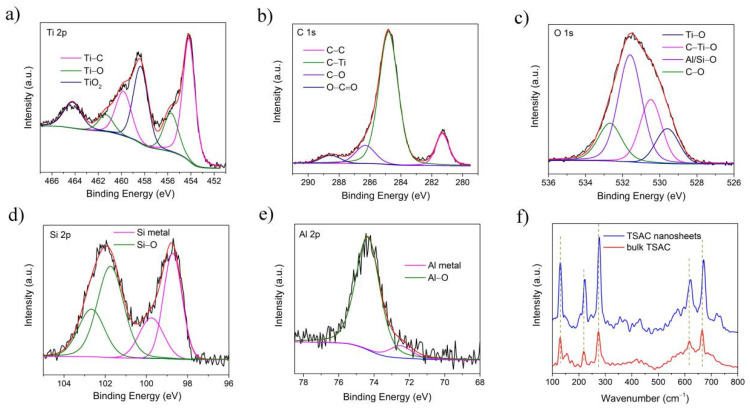
High resolution XPS spectra of TSAC nanosheets: (**a**) Ti 2p; (**b**) C 1s; (**c**) O1s; (**d**) Si 2p; (**e**) Al 2p; (**f**) Raman spectra of bulk TSAC and TSAC nanosheets.

Figure 4a–e show the high resolution XPS spectra of TSAC nanosheets. The XPS spectra of Ti 2p and C 1s are similar to those spectra of Ti_3_C_2_ MXene [24,26,27], which also includes Ti–C, Ti–O, TiO_2_, C–C, C–O and O–C=O bonds, indicating both Ti_3_C_2_ and TSAC nanosheets have similar intralayer and surface structure of MX layer. In addition, based on the existence of Ti–O, C–O, Si–O and Al–O bonds [28], it can be deduced there is an oxide layer covered on the surface of TSAC nanosheets, which is in agreement with previous reports of MAX phases [28]. In the spectra of Si 2p and Al 2p in Figure 4b,c, Si metal and Al metal bonds are detected [29], suggesting weak connections between the MX layer and the A layer in TSAC MAX phase nanosheets. Thus, bulk TSAC particles have the possibility to be exfoliated into thin nanosheets. The Raman spectra of bulk TSAC and TSAC nanosheets in Figure 4f further proved the layered structure of TSAC MAX phase, because both samples show distinct Raman peaks of Ti_3_SiC_2_ as in previous reports [30,31]. After delamination, these peaks of the received TSAC nanosheets exhibit small red shifts, which may be caused by the expanding crystal structure of the MAX phase [10]. Hence, by combining the results of SEM, TEM, XPS and Raman of TSAC nanosheets, it can be concluded that the exfoliated products are mainly composed of Ti_3_Si_0.75_Al_0.25_C_2_ phase and the layered structure of Ti_3_Si_0.75_Al_0.25_C_2_ phase has been well maintained during sonication.

**Figure 5:**
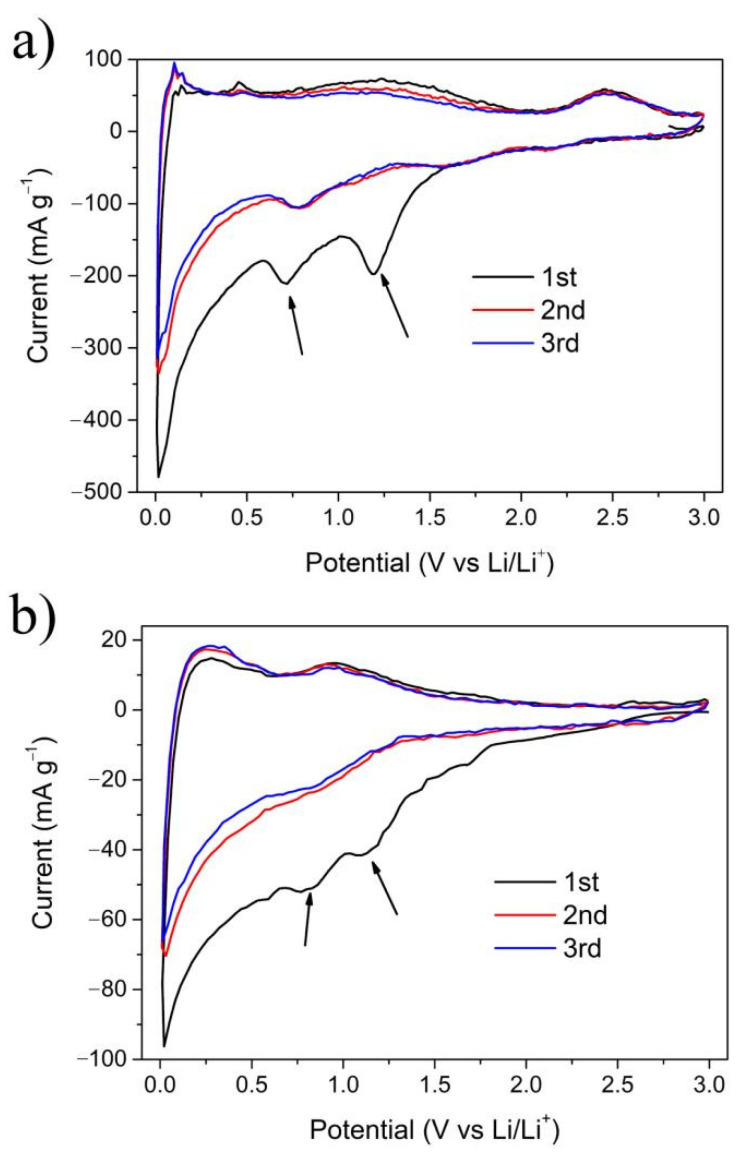
CV curves of (**a**) TSAC nanosheets and (**b**) bulk TSAC at a scan rate of 0.1 mV s^−1^.

The cyclic voltammetry (CV) and galvanostatic charge–discharge cycling (GV) of bulk TSAC and TSAC nanosheets were tested in this work. In Figure 5a,b, both TSAC nanosheets and bulk TSAC exhibit similar CV behaviors, except there is an additional cathodic peak of TSAC nanosheets around 2.4 V, showing that the exfoliated TSAC nanosheets perform a special Li^+^ deintercalation process. During the first reduction process of both electrodes, there is an obvious peak located at about 1.2 V associated with the generation of a solid electrolyte interphase (SEI) film [32]. This peak then practically disappears during the following reduction process, which can be ascribed to the isolation between the TSAC anode and electrolyte by the dense SEI film formed on the surface of the TSAC anode in the first reduction process. Then, a peak around 0.75 V is detected and kept at the following cycles, where the reaction of Li^+^ with TSAC nanosheets such as Ti_3_C_2_ probably occurs [32]. In the following cycles, two broad redox reversible peaks at 1.8 and 2.4 V corresponding to the reaction between Li^+^ and titanium oxide on the surface of TSAC nanosheets appear, because similar pairs were also observed in Ti_2_C and Ti_3_C_2_ nanosheets [32,33,34,35]. Interestingly, during the second and third cycles of TSAC nanosheets, a small cathodic peak at 0.1 V and two anodic peaks at 0.15 and 0.5 V can be observed, probably corresponding to lithiation process of Si and delithiation of Li_x_Si [36]. Meanwhile, for bulk TSAC, these redox peaks are not obvious because there are less exposed Si atoms in bulk TSAC particles. This alloying procedure of exposed “A” layer of MAX phases is also found in those Sn-containing MAX phases, such as Ti_2_SnC, Nb_2_SnC and V_2_SnC [7,8,9]. In addition, the differences in the CV profiles between these initial two cycles and the similarity in the second and the third cycles indicates that the irreversible capacity losses of TSAC nanosheets and bulk TSAC mainly take place in the first cycle.

**Figure 6:**
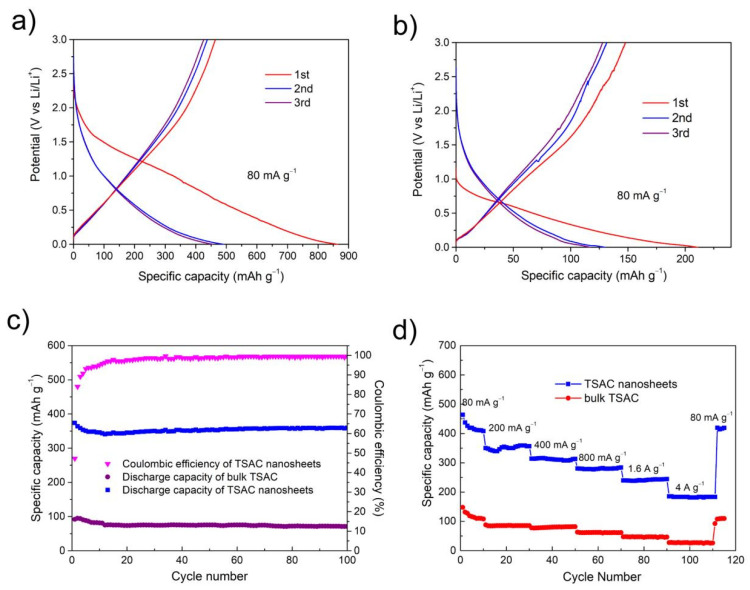
(**a**) The galvanostatic charge/discharge lithiation/delithiation curves of TSAC nanosheets; (**b**) the galvanostatic lithiation/delithiation curves of bulk TSAC; (**c**) specific delithiation (discharge) capacities of TSAC nanosheets and bulk TSAC vs. cycle number. The tests were carried at a current density of 200 mA g^−1^; (**d**) specific delithiation (discharge) capacities of TSAC nanosheets and bulk TSAC electrodes cycled at various current densities.

The CV profiles of TSAC nanosheets in Figure 5a also show no obvious charge and discharge capacity at potential higher than 3 V vs. Li/Li^+^. Thus, the lithium-ion storage tests for TSAC were performed from 0.001 to 3 V. The voltage profile for TSAC nanosheets at 80 mA g^−1^ (Figure 6a) delivers a beginning charge capacity (lithiation) of 862 mAh g^−1^. This capacity is much bigger than that of bulk TSAC with only 215 mAh g^−1^ (Figure 6b). It can also be detected from the charge profile in Figure 6a that more than three-quarters of the capacity are under 1.5 V, indicating that TSAC nanosheets could function better as anode materials for LIBs. Figure 6c shows the lithium-ion storage behavior of bulk TSAC and TSAC nanosheets with charge/discharge cycles at 200 mA g^−1^. The coulomb efficiency of TSAC nanosheets at the first cycle is 47.2%, which then increases rapidly and reaches 93.1% at the fifth cycle. The reason for the irreversibility in the initial cycles could be attributed to the formation of solid electrolyte interphase (SEI) or because of some irreversible reactions of Li ions with the surface groups and/or water molecules in TSAC nanosheets. On the whole, this irreversibility could be minimized by tailoring the surface structure of TSAC nanosheets or by prelithiating the electrode material as mentioned in other materials [37]. Then the reversible performance becomes stable after the first few lithiation/delithiation cycles. A stable cycle capacity of TSAC nanosheets is around 350 mAh g^−1^ at a current density of 200 mA g^−1^ and higher than the maximum theoretical capacity of f-Ti_3_C_2_ predicted by Tang et al. [38]. In addition, TSAC nanosheets show very good reversibility and stability, and a reversible capacity of 350 mAh g^−1^ is still kept after 100 cycles while it is only around 70 mAh g^−1^ for bulk TSAC.

**Figure 7:**
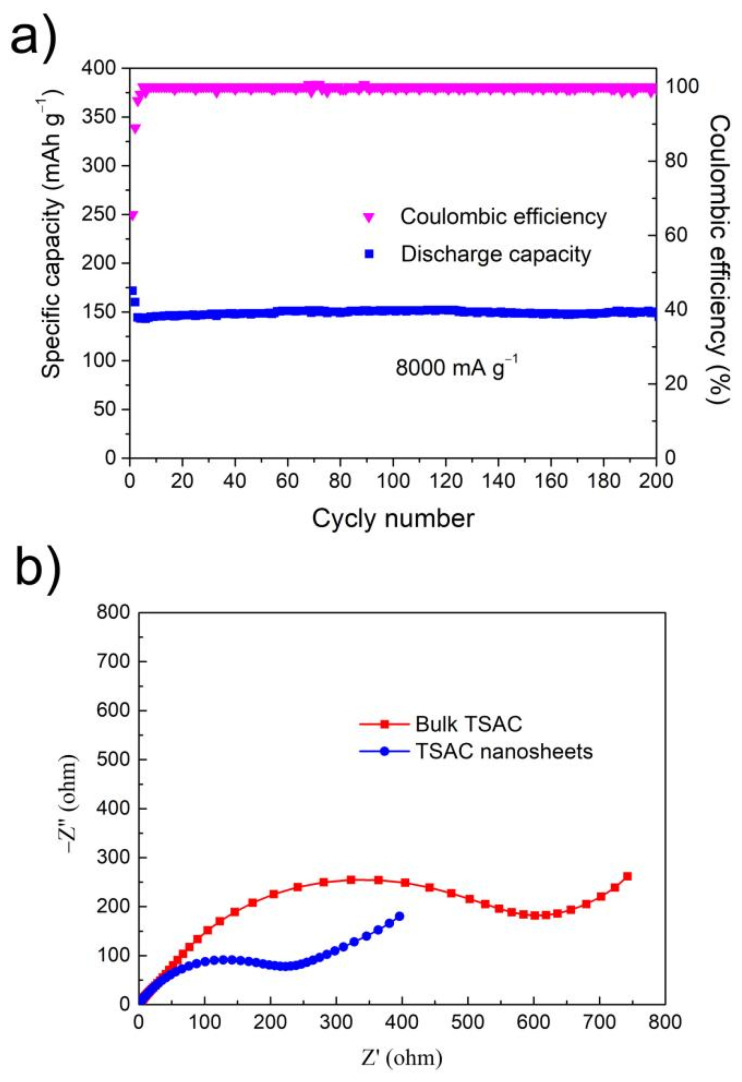
(**a**) Specific discharge capacity of TSAC nanosheets vs. cycle number at a current density of 8000 mA g^−1^; (**b**) Nyquist plots of bulk TSAC and TSAC nanosheets.

Rate-capability tests were also performed to further assess the electrochemical activities of TSAC nanosheets. As shown in Figure 6d, both TSAC nanosheets and bulk TSAC anodes manifest excellent cycling stability at various current densities. After 10 cycles at 80, 200, 400, 800, 1600 and 4000 mA g^−1^, TSAC nanosheets deliver delithiation capacities of 410, 353, 312, 279, 240 and 181 mAh g^−1^, while that of bulk TSAC is only 108, 85, 80, 62, 46 and 26 mAh g^−1^, respectively, suggesting a better rate performance for TSAC nanosheets at various rates. Even at a high rate of 8000 mA g^−1^, the TSAC nanosheets anode is capable of maintaining a discharge capacity of 150 mAh g^−1^ after 200 cycles (Figure 7a), which equals to that of the third cycle. In addition, the coulombic efficiency of TSAC nanosheets at 8000 mA g^−1^ is between 98–100% after the first five cycles, indicating a relatively stable SEI formation and negligible side reactions of the electrode. The superior rate performances suggest that TSAC nanosheets can be promising anode materials for LIBs, particularly in high-power applications. For instance, lithium titanate is well known due to its capability to handle high cycling rates, even at 10 C, and the capacity of Li_4_Ti_5_O_12_ is around 108 mAh g^−1^ [39]. Moreover, after hybridizing with graphene and Ag, high capacities of 133 and 156 mAh g^−1^ at 10 C are achieved for the resulting LTO/graphene and LTO/Ag, respectively [40,41]. In addition, MXenes with similar laminar structure are also capable of handling high cycling rates, such as V_2_CT_x_ (110 mAh g^−1^ at 10 C), Nb_2_CT_x_ (125 mAh g^−1^ at 10 C), annealed Nb_2_CT_x_ (342 mAh g^−1^ at 2 A g^−1^), f-T_3_C_2_ (110 mAh g^−1^ at 36 C) and porous T_3_C_2_T_x_ foam (101 mAh g^−1^ at 18 A g^−1^) [42,43,44,45].

Through Figure 6 and Figure 7, compared with bulk TSAC, TSAC nanosheets exhibited enhanced electrochemical properties as anode materials for LIB. According to the studies of lithium-ion uptake of other MAX phases, both MX and A layers show a possible redox reaction capability with lithium-ion [7,8,9]. In addition, after etching A layer from MAX phases, the resulting MXenes also show promising lithium-ion storage ability [44,46,47,48]. Compared to bulk TSAC, TSAC nanosheets can provide larger active surface area and more exposed Si atoms to promote the redox reactions of Ti_3_C_2_-Li and Si-Li; thus, the specific capacity of TSAC nanosheets is superior to that of bulk TSAC. The specific surface area (SSA) calculated using the BET equation for the TSAC nanosheets is 11.68 m^2^ g^−1^. This value is about three times higher than the bulk TSAC powders measured at around 4.25 m^2^ g^−1^. Moreover, TSAC nanosheets electrodes have better conductivity from their 2D graphene-like nanostructure, which can effectively promote the electron transfer and shorten the Li^+^ ions diffusion distance and the polarization, resulting in the improvement of their electrochemical performance. It is obvious that the semicircular arc of the electrochemical impedance spectroscopy (EIS) of TSAC nanosheets is smaller than that of bulk TSAC (Figure 7b), indicating that it has smaller charge transfer resistance. Specially, it can be anticipated that the electrochemical performance of TSAC nanosheets would be enhanced by optimizing and engineering the materials’ surfaces, structures and compositions, and/or by introducing additives as reported for other 2D materials, such as graphene [41,49,50,51], MoS_2_ [52,53,54] et al.

## 4. Conclusions

In this work, novel Ti_3_Si_0.75_Al_0.25_C_2_ ultrathin nanosheets as promising anode material for LIB are successfully developed by facile sonic exfoliating in alcohol. The nanosheets have a high capacity of ≈350 mAh g^−1^ at 200 mA g^−1^, high cycling stability and excellent rate performance (150 mAh g^−1^ after 200 cycles at 8000 mA g^−1^), which enhances the lithium-ion uptake capability in comparison with their bulk counterparts. It is noted that the reversible capacity of the nanosheets is about six times higher than the pristine bulk Ti_3_Si_0.75_Al_0.25_C_2_. In addition, more than 100 compounds have been found in the MAX family to date, and they all have similar potential due to their superior electrical conductivity, lamellar structure, very good stability even under severe environments and activated “A” layers for using as LIBs electrodes. Thus, our findings in this work are opening the door for the study on 2D MAX compounds as valuable LIBs electrodes, particularly for high power applications.

## Data Availability

No new data were created or analyzed in this study. Data sharing is not applicable to this article.
